# Prediction of Terpenoid Toxicity Based on a Quantitative Structure–Activity Relationship Model

**DOI:** 10.3390/foods8120628

**Published:** 2019-12-01

**Authors:** Rosa Perestrelo, Catarina Silva, Miguel X. Fernandes, José S. Câmara

**Affiliations:** 1 CQM, Centro de Química da Madeira, Universidade da Madeira, Campus da Penteada, 9020-105 Funchal, Portugal; cgsluis@staff.uma.pt; 2 BioLab, Instituto Universitario de Bio-Orgánica “Antonio González” (IUBO-AG), Universidad de La Laguna, C/Astrofísico Francisco Sánchez 2, 38200 La Laguna, Spain; mfernand@ull.edu.es; 3 Faculdade de Ciências Exatas e da Engenharia, Universidade da Madeira, Campus da Penteada, 9020-105 Funchal, Portugal

**Keywords:** terpenoids, *Vibrio fischeri*, toxicity, QSAR, heuristic method

## Abstract

Terpenoids, including monoterpenoids (C_10_), norisoprenoids (C_13_), and sesquiterpenoids (C_15_), constitute a large group of plant-derived naturally occurring secondary metabolites with highly diverse chemical structures. A quantitative structure–activity relationship (QSAR) model to predict terpenoid toxicity and to evaluate the influence of their chemical structures was developed in this study by assessing in real time the toxicity of 27 terpenoid standards using the Gram-negative bioluminescent *Vibrio fischeri*. Under the test conditions, at a concentration of 1 µM, the terpenoids showed a toxicity level lower than 5%, with the exception of geraniol, citral, (*S*)-citronellal, geranic acid, (±)-α-terpinyl acetate, and geranyl acetone. Moreover, the standards tested displayed a toxicity level higher than 30% at concentrations of 50–100 µM, with the exception of (+)-valencene, eucalyptol, (+)-borneol, guaiazulene, β-caryophellene, and linalool oxide. Regarding the functional group, terpenoid toxicity was observed in the following order: alcohol > aldehyde ~ ketone > ester > hydrocarbons. The CODESSA software was employed to develop QSAR models based on the correlation of terpenoid toxicity and a pool of descriptors related to each chemical structure. The QSAR models, based on *t*-test values, showed that terpenoid toxicity was mainly attributed to geometric (e.g., asphericity) and electronic (e.g., maximum partial charge for a carbon (C) atom (Zefirov’s partial charge (PC)) descriptors. Statistically, the most significant overall correlation was the four-parameter equation with a training coefficient and test coefficient correlation higher than 0.810 and 0.535, respectively, and a square coefficient of cross-validation (Q^2^) higher than 0.689. According to the obtained data, the QSAR models are suitable and rapid tools to predict terpenoid toxicity in a diversity of food products.

## 1. Introduction

Monoterpenoids (C_10_), norisoprenoids (C_13_), and sesquiterpenoids (C_15_) constitute a large group of plant-derived naturally occurring secondary metabolites with highly diverse chemical structures. They have various biological activities as well as a wide range of applications, including their use as agricultural products, flavorings, pharmaceuticals, and fragrances [1]. From a health point of view, terpenoids are known for their antibacterial, anticancer, anti-inflammatory, anthelmintic, antiviral, and antimalarial properties [2,3,4,5]. Terpenoids can function as antimicrobial agents to protect their natural hosts, with antibacterial activity occurring via disruption of the lipid membrane, resulting in the alteration of membrane organization and function [1,6]. As a result of lipophilic compounds partitioning into the lipid bilayer, damage occurs in the cell membrane by impairing vital functions (e.g., loss of ions, metabolites, lipids, and proteins; and dissipation of the pH gradient and electrical potential) [6,7,8]. Enzymes and DNA have also been mentioned as possible targets, as lipophilic compounds tend to associate with the hydrophobic core of several proteins leading to conformational changes, and consequently protein inactivation [6]. The toxicity level depends on the interaction with membrane constituents, concentration, and location. The accumulation of lipophilic compounds can occur at varying depths in the lipid bilayer. This depends on compound hydrophobicity as well as the influence of membrane composition, or the effect of external factors (e.g., temperature on terpene penetration ability) [8].

Several in vivo assays are available to measure chemical toxicity. Nevertheless, these experimental assays are expensive, labor-intensive, and time-consuming, which encourage the development of alternative more reliable, sensitive, and quick bioassays [9]. In recent years, a bioluminescence inhibition assay based on *Vibrio fischeri* (Gram-negative bacterium) has been widely used to perform toxicity measurements. This assay showed good reproducibility, sensitivity, cost-effectiveness, and ease of operation and is an efficient ethical alternative to testing on higher species [10]. Researchers have reported the *V. fischeri* bioluminescence assay as the most sensitive across a wide range of chemicals compared to other bacterial assays, such as nitrification inhibition, respirometry, adenosine triphosphate (ATP) luminescence, and enzyme inhibition [11,12]. This strain is also commercially available in several test kits, i.e., Microtox, Aboatox, LUMIStox, and ToxAlert [13].

The quantitative structure–activity relationship (QSAR) analysis is usually used to develop mathematical models that relate small variations of chemical structure, parameterized by empirical physicochemical or theoretical molecular descriptors, to biological activity [14]. Different types of numerical molecular descriptors have been employed, which are related to constitutional, topological, geometrical, electronic, and quantum chemical origins [15]. Nevertheless, several steps should be taken into consideration to develop a robust and sensitive QSAR model, such as (i) understanding the interaction mechanism between chemical and biological systems, (ii) selection of a relevant descriptor set that describes the relationship between the chemical and activity/property under consideration, and (iii) selection of statistical tools [15,16,17].

Some studies have been performed on the relationship between toxicity and chemical structure of several compounds, and QSARs models have been developed to predict *V. fischeri* toxicity for specific groups using molecular and physicochemical descriptors [18,19,20,21,22,23]. The toxicity of narcotic compounds against *V. fischeri* was predicted using molecular connectivity indices (topological descriptors), and the data obtained suggested that the degree of branching and the compounds’ electronic characteristics have a dominant role in the toxicity level [20]. Topological, electronic, and log P descriptors have also been used to predict the toxicity of organic pollutants against *V. fischeri* [19]. Charge distribution (e.g., maximum partial charge for a carbon (C) atom (Zefirov’s partial charge (PC)) and geometric (e.g., shadow parameter) descriptors were used by Couling et al. [21] to assess the toxicity of a diversity of ionic liquids against *V. fischeri* and *Daphnia magna*. In addition, Das et al. [18] developed predictive QSAR models for the ecotoxicity of ionic liquids using the bacteria *V. fischeri* as an indicator response species. Regarding terpenoids toxicity, Vinholes et al. [24] evaluated the hepatoprotection effect of fifteen sesquiterpenoids with different chemical structures, commonly found in plants and plant-derived foods and beverages, using a QSAR approach. With the exception of α-humulene, all the sesquiterpenoids under study (1 mM) were effective in reducing the malonaldehyde levels in both endogenous and induced lipid peroxidation up to 35% and 70%, respectively [24]. QSAR studies were performed in order to predict the insecticidal activity of terpene compounds, since insects affect food production and human health (Dambolema 2016). Grodnitzky and Coats [25] developed effective models to explain and predict the insect toxicity of monoterpenoids and their derivatives, and the results showed that thymol and two ether derivatives had the greatest toxicity to the house fly. Moreover, Chang et al. [26] developed a QSAR model to study the neuroprotective activity of thirteen terpenoids on human neuroblastoma SH-SY5Y cells, and *trans*-caryophyllene turned out to be the most promising neuroprotective terpene among the thirteen terpenoids tested. The aim of the current study was to evaluate the toxicity of 27 terpenoids (16 monoterpenoids, 8 sesquiterpenic compounds, and 3 norisoprenoids) at different concentrations (1, 10, 50, and 100 µM) and incubation times (0, 20, 40, 60, 80, and 100 min) using the *V. fischeri* bioluminescence inhibition assay. The previous experimental data set obtained was then used to develop QSAR models using the CODESSA (comprehensive descriptors for structural and statistical analysis) software to predict the terpenoid-related chemical structure toxicity.

## 2. Materials and Methods

### 2.1. Reagents

Ethanol (99.9%), potassium dihydrogen phosphate (KH_2_PO_4_, 99%), glycerol (87%), peptone from casein, meat extract, and tryptic soy agar (TSA) were obtained from Merck (Darmstadt, Germany). Agar was obtained from Liofilchem (Teramo, Italy). Anhydrous sodium carbonate (99.8%), sodium chloride (NaCl, 99%), sodium hydroxide (NaOH, 98%), and potassium chloride (KCl, 99%) were purchased from Panreac (Barcelona, Spain) and sodium dihydrogen phosphate dihydrate (Na_2_HPO_4_ 2H_2_O, 99%) was obtained from Fluka (Buchs, Switzerland).

### 2.2. Terpenoids Standards

**Figure 1:**
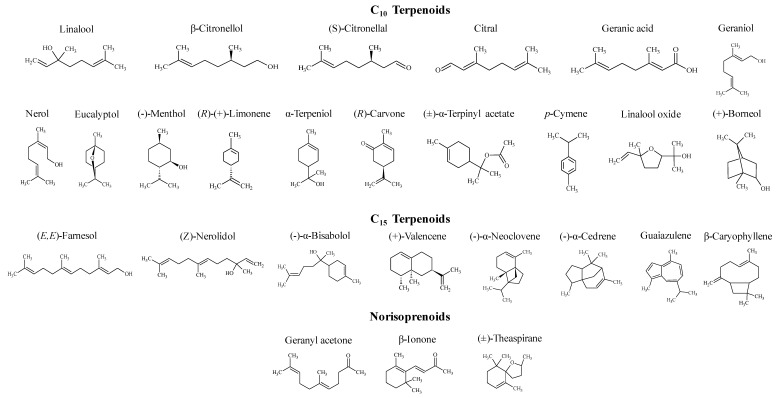
Chemical structures of terpenoids.

Figure 1 shows the chemical structures of terpenoids used as authentic standards to evaluate toxicity. Nerol (90%), β-caryophyllene (98.5%), (-)-α-cedrene (99%), (-)-α-neoclovene (95%), (+)-valencene (70%), (*Z*)-nerolidol (95%), and (-)-α-bisabolol (95%) were purchased from Fluka (Buchs, Switzerland). *p*-Cymene (99%), (*R*)-(+)-limonene (97%), (+)-borneol (97%), eucalyptol (99%), geraniol (98%), linalool (98.5%), α-terpeniol (95%), β-citronellol (95%), (-)-menthol (99%), (*R*)-carvone (98%), citral (95%), (*S*)-citronellal (96%), geranic acid (85%), linalool oxide (97%), (±)-α-terpinyl acetate (90%), β-ionone (97%), geranyl acetone (98%), (±)-theaspirane (90%), and (*E*,*E*)-farnesol (96%) were obtained from Sigma-Aldrich Química S.A. (Madrid, Spain). Guaiazulene (98%) was purchased from TCI Europe N.V. (Zwijndrecht, Belgium). For each terpenoid standard, an ethanolic stock solution was prepared (50 mM). From each stock solution, working solutions were prepared by diluting adequate amounts in order to obtain a final concentration of 0.2, 1, 10, and 20 mM. All the solutions were stored at −20 °C.

### 2.3. Assessment Terpenoid Toxicity

*V. fischeri* terpenoid exposures were conducted according to the methodology previously described [27]. The bioluminescent marine bacterium *V. fischeri* ATCC 49387 (USA) was used. It produces light without the addition of exogenous substrates, and the light emission is directly proportional to its metabolic activity. Fresh plate cultures of bioluminescent *V. fischeri* were maintained in solid BOSS medium (1% peptone, 0.3% beef extract, 0.1% glycerol, 3% NaCl, 1.5% agar, pH 7.3) at 4 °C. An NaCl concentration range from 20 to 40 g/L is needed to maintain the osmotic pressure of cells that is required for natural light emission to occur. Before each bioassay, one isolated colony was aseptically inoculated in 30 mL of liquid BOSS medium, and grown for 16 h at 25 °C under constant stirring (120 rpm). An aliquot of this culture (240 µL) was subcultured in 30 mL of BOSS medium, and grown overnight at 25 °C under stirring (120 rpm) to reach an optical density (OD_620_) of ≈1.0, corresponding to ≈10^8^ CFU/mL. For the bioassays, an overnight culture of *V. fischeri* was used after a ten-fold dilution in phosphate-buffered saline (PBS: 30 g NaCl, 0.2 g KCl, 1.44 g Na_2_HPO_4_, and 0.24 g KH_2_PO_4_ per liter; pH 7.4) to achieve a final concentration of 10^7^ CFU/mL.

For each terpenoid experiment, 10 mL of bacterial suspension was aseptically distributed in 100 mL acid-washed, sterilized, glass beakers and 50 µL working stock solution of each standard (0.2, 2, 10, and 20 mM in hydroalcoholic solution) was added in order to achieve a final concentration of 1, 10, 50, and 100 μM, respectively. Then, all the beakers were wrapped with aluminum foil to protect from light exposure and incubated under stirring (120 rpm) at 20–25 °C. A control experiment, consisting of bacterial suspension and hydroalcoholic solution, instead of terpenoids, was carried out simultaneously. Aliquots of 500 µL of the standard C_10_ and C_15_ terpenic compounds and norisoprenoids and the control were collected at different times (0, 20, 40, 60, 80, and 100 min) and the bioluminescence signal (peak wavelength detected at 420 nm, standard range 300–650 nm) was measured on a luminometer (TD-20/20 Luminometer, Turner Designs, Inc., Sunnyvale, CA, USA). Three independent assays were performed for each component and the control and the results were averaged.

### 2.4. Calibration of Bioluminescent Signal and Viable Cell Numbers

The correlation between the colony-forming units (CFU) and the bioluminescent signal (in relative light units, RLU) of *V. fischeri* was performed. For this purpose, eight-fold serial dilutions of the culture were prepared in PBS with 3% NaCl. The non-diluted (100) and diluted aliquots were spread-plated in tryptic soy agar (TSA) with 3% NaCl (100 µL) to determine the number of viable cells (CFU/mL), and simultaneously bioluminescence was read on the luminometer (500 µL) to determine the bioluminescence signal. Both experiments were performed in triplicate and the results were averaged. The toxicity result of each terpenoid concentration at different incubation times was calculated as percentage inhibition, relative to the control sample (0 min), through the following equation:

% Inhibition = ((Gc − Gs)/Gc) ×100

where Gc specifies the arithmetic mean of the bioluminescence values of the control and Gs indicates the bioluminescence value of a particular sample after incubation times.

### 2.5. QSAR Model Development

#### 2.5.1. Geometry Optimization and Calculation of Molecular Descriptors

The three-dimensional chemical structures of the terpenoids were drawn and pre-optimized using the AMBER force field model available in the HYPERCHEM 7.0 software (Hypercube Inc, Gainsville, FL, USA). The final molecular geometries were refined using the quantum chemical program package MOPAC 6.0 (Chicago, IL, USA). The Austin Model 1 (AM1) parameterization with eigenvector following the geometry optimization procedure at a precision level 0.01 kcal/Å gradient norm was used to calculate electronic and thermodynamic descriptors.

The CODESSA software (Semichem Inc, Shawnee, KS, USA) was used to calculate a pool of different molecular descriptors using the MOPAC output files, HyperChem structure files, and additional descriptors calculated using the DRAGON software package (Chicago, IL, USA) [28]. In total, more than 280 molecular descriptors were generated for each structure, which could be organized into five groups, namely constitutional, topological, geometrical, electrostatic, and quantum chemical. These molecular descriptors contain information about the connections, shape, symmetry, charge distribution, and quantum chemical properties of the chemical structures under study.

#### 2.5.2. Development and Validation of QSAR Models

An important step in QSAR model development is the selection of the best multilinear regression equation among a given descriptor set. Once molecular descriptors are calculated, the selection was performed using the heuristic method (HM) available in the framework of the CODESSA software, which reduces the descriptors pool by eliminating descriptors (i) that are not available for all the structures studied, (ii) that have a constant value for all the structures studied, (iii) with F-values below 1, (iv) with *t*-test values lower than 0.1 at a probability level of 0.05, and (v) that are highly correlated and provide approximately identical information, if their pair-wise correlation coefficient exceeds 0.80 [29]. The selected descriptors were then used for developing the QSAR prediction models by multiple linear regressions (MLRs), with a training subset composed of 22 terpenoids. The predictive power of the resulting models was evaluated by a test subset of five terpenoids representative of the biological activity of data set. For the training subset of the 22 terpenoids, not more than four descriptors were considered for the correlation analysis, thereby keeping the ratio to a maximum of 4:1 [30].

The QSAR models derived from MLR analyses were then used in a validation study (in order to select the reliable and robust models) by taking into account the highest squared correlation coefficient (*r*^2^), square coefficient of cross validation (Q^2^), Fisher F-criterion value (ratio of regression and residual variances and reflects the significance of the model), and Student’s *t*-test (reflects the significance of the parameter within the model), as well as the lowest standard deviation (S). Generally, Q^2^ is used as a criterion for both the robustness and predictive ability of QSAR models. Many researchers considered high Q^2^ (for instance, Q^2^ higher than 0.50) as an indicator or even as the ultimate proof of the high predictive power of QSAR models [28,31].

## 3. Results and Discussion

### 3.1. Toxicity of Terpenoids

**Figure 2:**
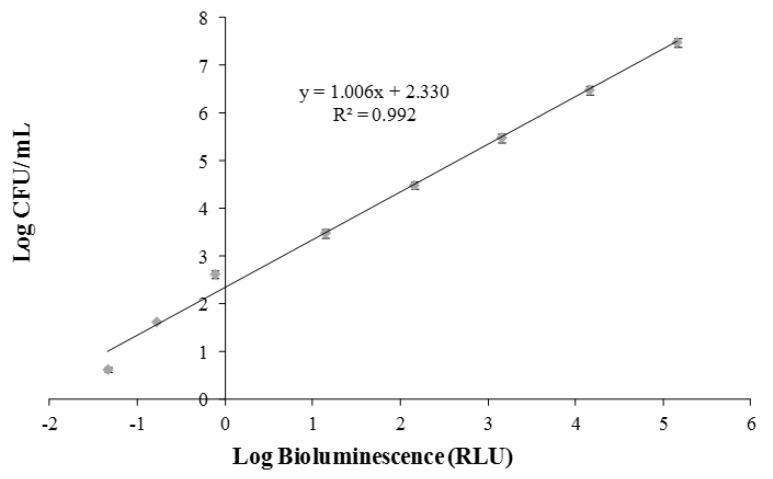
Relationship between the bioluminescence signal and viable counts of an overnight culture of *Vibrio fischeri* (≈10^9^ CFU/mL) serially diluted in phosphate buffer solution (PBS) with 3% NaCl. Bioluminescence is expressed in relative light units (RLU) and viable counts in CFU/mL. Values represent the mean of two independent experiments; error bars indicate the standard deviation.

**Figure 3:**
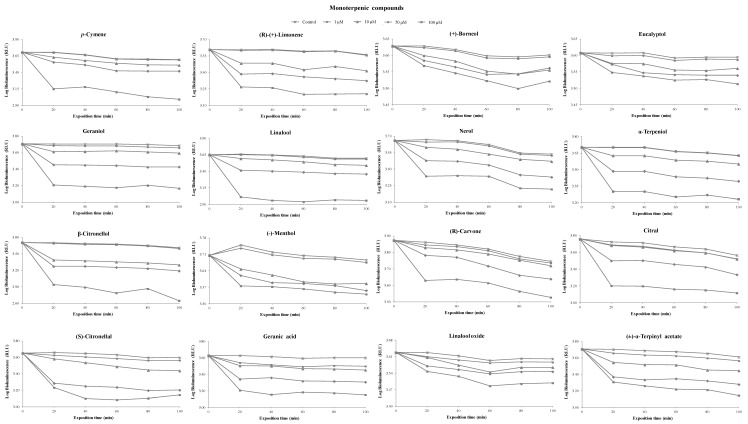
Bioluminescence monitoring of *V. fischeri* exposed to monoterpenic compounds at different concentrations. The values are expressed as the means of three independent experiments.

**Figure 4:**
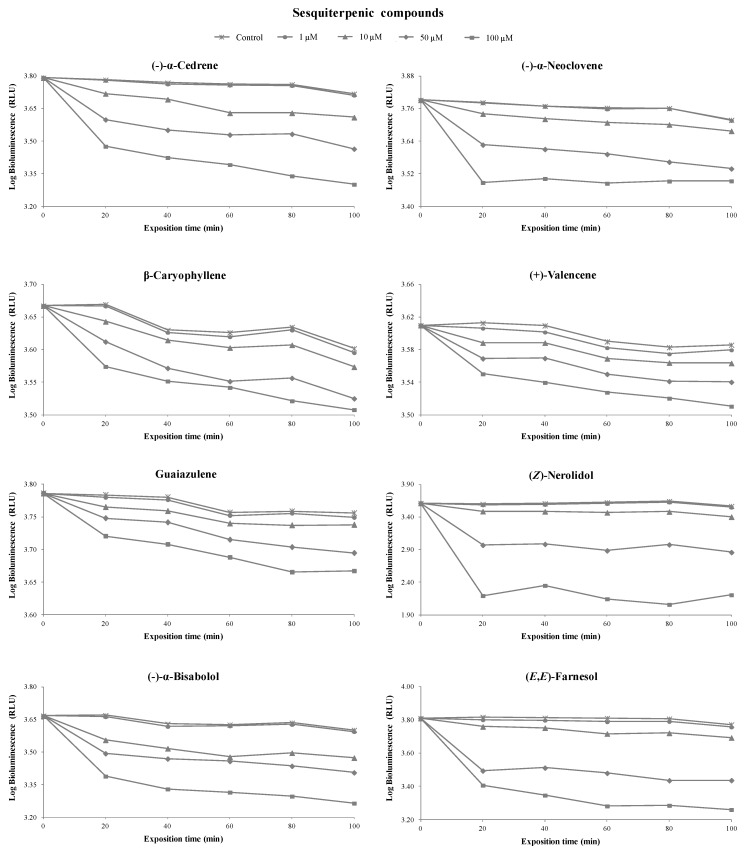
Bioluminescence monitoring of *V. fischeri* exposed to sesquiterpenic compounds at different concentrations. The values are expressed as the means of three independent experiments.

**Figure 5:**
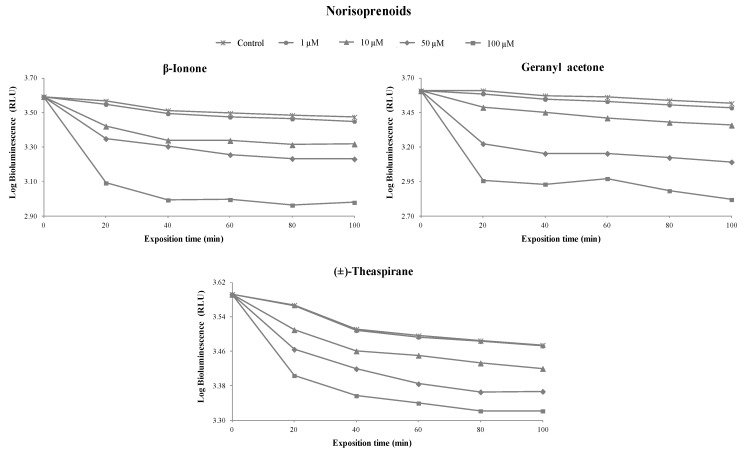
Bioluminescence monitoring of *V. fischeri* exposed to norisoprenoids at different concentrations. The values are expressed as the means of three independent experiments.

A correlation between the colony-forming units (CFU) and the bioluminescent signal (in relative light units, RLU) of overnight cultures of the *V. fischeri* bioluminescent strain was performed to evaluate the viable bacterial abundance. A linear correlation, reflecting the viable bacterial abundance, was observed (Figure 2). This section is divided by subheadings. It should provide a concise and precise description of the experimental results and their interpretation, as well as the experimental conclusions that can be drawn. Figure 3 Figure 4 Figure 5 and Tables S1–S3 show the inhibitory percentage of *V. fischeri* exposed to 27 terpenoids (16 monoterpenoids, 8 sesquiterpenoids, and 3 norisoprenoids) at different concentrations (1, 10, 50, and 100 µM) and incubation times (0, 20, 40, 60, 80, and 100 min).

At the concentration of 1 µM, geranic acid (20%), (±)-α-terpinyl acetate (11%), citral (9%), and (*S*)-citronellal (8%) showed a higher toxicity level than the remaining terpenoids tested. At the concentration of 10 µM, β-citronellol (52%) showed a considerable toxicity level, followed by (±)-α-terpinyl acetate (32%), β-ionone (31%), geranyl acetone (28%), (*Z*)-nerolidol (28%), limonene (28%), geranic acid (26%), (-)-α-bisabolol (26%), and (*S*)-citronellal (25%). The remaining terpenoids under study showed toxicity lower than 21%. The results showed that toxicity was proportional to standard concentration. At the concentration of 100 µM, the majority of terpenoids tested showed toxicity higher than 50%, with the exception of (+)-valencene (14%), eucalyptol (15%), (+)-borneol (16%), guaiazulene (16%), β-caryophellene (19%), linalool oxide (20%), (-)-menthol (29%), (+)-theaspirane (30%), (*R*)-carvone (39%), and (-)-α-neoclovene (46%).

Regarding the incubation time, no remarkable differences were observed between 20 and 100 min, indicating that greater toxicity occurred during the first 20 min. For this reason, this incubation time was selected to develop the QSAR models to predict terpenoid toxicity. An overview could be achieved based on the relationship between the toxicity of terpenoids and their chemical structures, as well as their functional groups. 

Concerning the functional groups, the highest toxicity level was observed in the following order: alcohol (e.g., geraniol) > aldehyde (e.g., (*S*)-citronellal) ~ ketone (e.g., geranyl acetone) > ester (e.g., (±)-α-terpinyl acetate) > hydrocarbons (e.g., (+)-valencene). The toxicity of some C_10_ and C_15_ terpenoid alcohols has been previously reported [32]. The presence of hydroxyl groups is crucial to the toxicity level, suggesting that the binding sites may contain both hydrogen bond donors as well as hydrogen bond receptors [33]. This could be confirmed by comparing the toxicity of α-terpineol with that of eucalyptol. The toxicity of chemical structures of terpenoids could also increase due to the presence of an oxygen-related function (e.g., geranyl acetone, β-ionone, (*S*)-citronellal, and citral). The presence of these functional groups increases the structure electronegativity, which may interfere with biological processes involving electron transfer and react with vital nitrogen components, such as proteins and nucleic acids, and consequently inhibit bacterial growth [34]. Moreover, monoterpenes consisting of aldehydes possess antimicrobial activity, which can be explained through their carbon double bond arrangements creating high electronegativity (reviewed by Mahizan et al. [35]). The presence of the acetate moiety in terpenoid chemical structures is also crucial to increase toxicity, which was confirmed when the activity of α-terpineol against *V. fischeri* was compared to (±)-α-terpinyl acetate (Figure 3). Similar results were reported for geraniol and (+)-borneol, where their toxicity was lower than the acetates against a diversity of bacteria [34]. The toxicity of geraniol has been investigated in several organisms and geraniol showed several biological properties, including antimicrobial, antioxidant, and anti-inflammatory activities, together with negligible toxicity (reviewed by Chen at al. [36]). The bacterial activity also depends on the alkyl substituent on the ring structure, which could be confirmed when the antibacterial activity of (*R*)-(+)-limonene (alkenyl substituent) was compared with that of *p*-cymene (alkyl substituent). The presence of a double bond in the chemical structures of C_10_ and C_15_ terpenoids and norisoprenoids contributed to an increase in their toxicities.

Finally, the terpene hydrocarbons (e.g., (+)-valencene, guaiazulene, and β-caryophyllene) showed low toxicity compared to the other terpenoids in the study. This could be explained by their low water solubility that limits their diffusion through the medium. This data are in agreement with a previous study, in which the C_10_ and C_15_ terpene hydrocarbons were relatively inactive independent of their chemical structure, due to their limited hydrogen capacity and water solubility [37]. The action sites of the terpene hydrocarbons appeared to be at the lipid bilayer, caused by biochemical mechanisms catalyzed by the lipid bilayers of the cells. These processes included the inhibition of electron transport, protein translocation, phosphorylation steps, and other enzyme-dependent reactions.

### 3.2. QSAR Models to Predict the Toxicity of Terpenoids

The heuristic method (HM) was applied to generate QSAR models with four descriptors. A subset composed of 22 terpenoids was built and the remaining five terpenoids were used in an external validation subset. The HM results are shown in Table 1. The QSAR models performed well, with a training correlation coefficient (*r*^2^_training_) and a test correlation coefficient (*r*^2^_test_) subset higher than 0.810 and 0.535, respectively. The square coefficient of cross validation (Q^2^) values were higher than 0.689, which suggested a high predictive power. Although, the QSAR models developed to predict the terpenoids’ chemical structure-related toxicity were characterized by good statistical parameters, such as *r*^2^_training_, *r*^2^_test_, and Q^2^, a good QSAR model fit depends on the experimental data quality. An extreme outlier was found in the QSAR model generated for the terpenoids at the concentration of 10 µM, and for this QSAR model, (*Z*)-nerolidol (outlier) was removed in order to improve the statistical result (Table 1). The four descriptors involved in the QSAR models obtained for the different concentrations of each terpenoids are listed in Table 1, and included constitutional, topological, geometrical, electrostatic, and quantum chemical descriptors.

For the terpenoid concentration 1 µM (equation 1), the QSAR model was constituted by two electronic (maximum partial charge for a C atom (Zefirov’s PC), Q^c^_max_; and WNSA-1 weighted PNSA (PNSA1×TMSA/1000) (Zefirov’s PC)), one topological (Kier shape index 3rd order, ^3^κ), and one quantum chemical (maximum atomic orbital electronic population, Max-OP) descriptors. According to the *t*-test, the most significant descriptor to predict terpenoid toxicity was Q^c^_max_, followed by Max-OP, ^3^κ, and WNSA-1. Q^c^_max_ is an electronic descriptor calculated from Zerirov’s electronegativity equation, and describes the most positively charged C atom in a molecule that is usually connected to an electron-withdrawing functional group or atom [38]. The Max-OP descriptor for a given atomic species in a molecule is a simplified index to describe the nucleophilicity of the molecule and could be interpreted as its ability to undergo oxidation and start a degenerative metabolic process [39]. The negative coefficient obtained for this quantum chemical descriptor indicated that the toxicity of the terpenoids increased with a decrease in the Max-OP magnitude.

In ^3^κ, the shape of a molecule depends on the number of skeletal atoms, molecular branching, and the ratio of the atomic radius and the radius on the carbon atom in the sp^3^ hybridization state [38]. The positive coefficient of the ^3^κ descriptor suggested that the increase in molecule branching and the presence of heteroatoms promoted terpenoid toxicity. The WNSA-1 descriptor describes the negative partial charge distribution information in a molecule and then could account for the electrostatic interaction between the compound and its receptor [38]. A negative coefficient of the WNSA-1 descriptor implies that the activity increases as the value of this descriptor decreases. As observed, the electronic descriptors involved in this model indicated that they are charged partial surface area (CPSA) descriptors, which suggested that surface area alone, as a geometric descriptor, was not sufficient to predict terpenoid toxicity. This is in agreement with previous studies that used CPSA descriptors to assess molecule lipophilicity [30].

For the terpenoid concentration 10 µM (equation 2), the QSAR model was constituted by one geometric (asphericity), two electronic (Q^c^_max_, PNSA-1 partial negative surface area (Zefirov’s PC)), and one physicochemical (log P) descriptors. According to the *t*-test, these descriptors obey the following order of significance: asphericity > PNSA-1 > Q^c^_max_ ~ log P. Asphericity (Ω) is a geometric descriptor which describes a molecule’s deviation from the spherical shape, and calculated from the eigenvalue λ_i_ of the inertia matrix [40]. The positive sign of asphericity indicated that terpenoid toxicity was promoted by linear (Ω = 1) and oblate (Ω ~ 1) structures of the molecules. PNSA-1 describes the sum of the surface area of negative atoms, and as observed for the first model (equation 1, Table 1), the negative sign of this electronic descriptor highlighted that a decrease in the magnitude of PNSA favored terpenoid toxicity. Again, Q^c^_max_ showed a positively correlation with terpenoid toxicity. This result is in agreement with literature as the Gram-negative outer layer membrane is composed primarily by lipopolysaccharide molecules, and forms a hydrophilic permeability barrier providing protection against the effects of highly hydrophobic compounds [33].

At 50 µM terpenoid concentration (equation 3), the QSAR model was also constituted by one geometric (asphericity), one topological (Kier and Hall index 2nd order, ^2^χ^υ^), one electronic (PNSA-1), and one quantum chemical (minimum atomic orbital electronic population, Min-OP) descriptors. According to the *t*-test, these descriptors obey the following order of significance: asphericity > ^2^χ^υ^ > PNSA-1 > Min-OP (equation 3, Table 1). As observed in QSAR model 2, asphericity and PNSA-1 showed a positive and negative correlation, respectively, with terpenoid toxicity. ^2^χ^υ^ is a valence connectivity topological descriptor, which reflects the branching molecule and also encodes the molecule size. The positive sign of this descriptor indicated that high molecular branching promoted less London dispersion, consequently increasing terpenoid toxicity. The Min-OP descriptor for a given atomic species in a molecule is a simplified index to describe the electrophilic ability of the molecule and connected to the hydrogen donor capabilities of the molecule.

At 100 µM terpenoid concentration (equation 4, Table 1), based on *t*-test, the most significant descriptor in this model affecting terpenoid toxicity was asphericity followed by XY shadow, number of single bonds (C_1_), and ^2^χ^υ^, which indicated that toxicity was affected by geometric, topological, and constitutional descriptors, but not by any electrostatic or quantum chemical descriptor. Again, asphericity showed a positive correlation with terpenoid toxicity, which indicated that toxicity was favored by an increase in asphericity magnitude, as observed in QSAR models 2 and 3 (Table 1). XY shadow is defined as the area of shadows of a molecule as projected on the XY plane by the orientation of the molecule in the space along the axes of inertia, which characterizes the size and geometrical shape of the molecule. Thus, it can act as a descriptor of van der Waals and dispersion interactions between the chemical compound and lipids [41].

The QSAR models generated for each concentration suggested that the charge distribution over the molecule as well as shape, size, and orientation of substituents remarkably influenced terpenoid toxicity. Moreover, it can be concluded that the developed models corresponding to the terpenoid concentrations of 10, 50, and 100 µM followed the same tendency, as according to the *t*-test values, the toxicity was mainly affected by steric effects (e.g., asphericity), with β-citronellol (Ω = 0.71), (*E*,*E*)-farnesol (Ω = 0.69), (*S*)-citronellal (Ω = 0.68), geranic acid (Ω = 0.63), (*Z*)-nerolidol (Ω = 0.63), geranyl acetone (Ω = 0.61), citral (Ω = 0.61), (-)-α-bisabolol (Ω = 0.60), geraniol (Ω = 0.58), nerol (Ω = 0.57), linalool (Ω = 0.56), and (±)-α-terpinyl acetate (Ω = 0.56) exhibiting the most toxicity. In sum, the presence of hydroxyl groups as well as oxygen-related functions are crucial to terpenoid toxicity levels, since the presence of these functional groups increases structure electronegativity, which may interfere with biological processes involving electron transfer and react with vital nitrogen components (e.g., proteins and nucleic acids). Other QSAR studies revealed that the number of conjugated carbons, the number of phenolic and hydroxyl groups, and the number of acceptor atoms of hydrogen bonds are the most important structural descriptors in the antimycobacterial activity of terpenes [42].

## 4. Conclusions

The current study reports the toxicity of terpenoids against *V. fischeri* bacteria. Concerning the functional groups, terpenoid toxicity decreased in the following order: alcohol (e.g., geraniol) > aldehyde (e.g., (*S*)-citronellal) ~ ketone (e.g., geranyl acetone) > ester (e.g., (±)-α-terpinyl acetate) > hydrocarbons (e.g., (+)-valencene). The high sensibility of *V. fischeri* to the cytotoxic effect of terpene alcohols could be explained by the involvement of the hydroxyl group in the formation of hydrogen bonds with the membrane polar part, whereas the low sensibility of *V. fischeri* to the cytotoxic effect of hydrocarbon terpenes could be explained by the fact that the Gram-negative outer layer membrane is primarily composed of lipopolysaccharide molecules and forms a hydrophilic permeability barrier providing protection against the effects of highly hydrophobic compounds.

The previous experimental data set was used to generate the QSAR models. The models performed well, with high significant correlation obtained using the heuristic method indicating that a combination of different molecular descriptor types resulted in the best correlation which could be used to predict the chemical structure-related terpenoid toxicity. Among the obtained models, several common descriptors were found, including two electronic (maximum partial charge for a C atom (Zefirov’s PC) and PNSA-1 partial negative surface area (Zefirov’s PC)), one geometric (asphericity), and one topological (Kier and Hall index 2nd order) descriptors. Their statistical significance depended on terpenoid concentration; for the lowest concentration (1 µM) tested, the most significant was an electronic descriptor (maximum partial charge for a C atom (Zefirov’s PC)), whereas for the remaining tested concentrations, the most significant was a geometric (asphericity) descriptor. Both the descriptors showed a positive correlation with toxicity, suggesting that molecule branching, the presence of heteroatoms, and electronegativity play a dominant role in terpenoid toxicity, and the most potentially toxic terpenoids were β-citronellol, (*E*,*E*)-farnesol, (*S*)-citronellal, geranic acid, (*Z*)-nerolidol, (-)-β-bisabolol, geraniol, nerol, linalool, geranyl acetone, β-ionone, citral, geranic acid, and (±)-α-terpinyl acetate. The developed QSAR models provided suitable and rapid tools to predict terpenoid toxicity present in a diversity of food products, in terms of antimicrobial activity.

## Figures and Tables

**Table 1:** Quantitative structure–activity relationship (QSAR) models obtained for the different concentrations of terpenoids against *V. fischeri* bacteria for an exposition time of 20 min.

(Terpenoids) (µM)	Nº	B	*t*-Test	Molecular Descriptors	Statistical Parameters
1	0	18.36	8.56	Intercept	*r*^2^_training_ = 0.952
	1	209.81	14.73	Maximum partial charge for a C atom (Zefirov’s PC)	*r*^2^_test_ = 0.923
	2	−10.28	−8.28	Maximum atomic orbital electronic population	F = 84.14
	3	0.93	6.70	Kier shape index (3rd order)	s^2^ = 1.05
	4	−0.11	−3.90	WNSA-1 weighted PNSA (PNSA1×TMSA/1000) (Zefirov’s PC)	Q^2^ = 0.900
10^a^	0	31.85	7.68	Intercept	*r*^2^_training_ = 0.873
	1	42.92	8.06	Asphericity	*r*^2^_test_ = 0.6987
	2	−0.30	−6.50	PNSA-1 partial negative surface area (Zefirov’s PC)	F = 27.57
	3	148.82	4.50	Maximum partial charge for a C atom (Zefirov’s PC)	s^2^ = 11.25
	4	−3.92	−4.48	Log P	Q^2^ = 0.794
50	0	39.71	1.07	Intercept	*r*^2^_training_ = 0.810
	1	110.40	7.40	Asphericity	*r*^2^_test_ = 0.535
	2	7.53	3.71	Kier and Hall index (2nd order)	F = 18.17
	3	−0.28	−2.81	PNSA-1 partial negative surface area (Zefirov’s PC)	s^2^ = 62.05
	4	−77.99	−1.95	Minimum atomic orbital electronic population	Q^2^ = 0.689
100	0	19.48	0.94	Intercept	*r*^2^_training_ = 0.846
	1	195.98	8.58	Asphericity	*r*^2^_test_ = 0.676
	2	21.01	0.21	Kier and Hall index (2nd order)	F = 23.39
	3	−1.17	−4.54	XY shadow	s^2^ = 103.69
	4	−72.04	3.96	Relative number of single bonds	Q^2^ = 0.734

^a^ N_training_ = 21 (training group), N_test_ = 5 (test group); Nº—number of descriptors; B—equation coefficient; *r*^2^_training_—training correlation coefficient; *r*^2^_test_—test correlation coefficient; F—Fisher F-criterion value; s^2^—Student’s *t*-test; Q^2^—square coefficient of cross validation; WNSA—weighted negative surface area; PNSA—partial negative surface area; TMSA—total molecular surface area.

## Supplementary Materials

The following are available online at https://www.mdpi.com/2304-8158/8/12/628/s1, Table S1: Monoterpenic compounds toxicity expressed as percentage (%) at different concentrations, Table S2: Sesquiterpenic compounds toxicity expressed as percentage (%) at different concentrations, Table S3: Norisoprenoids toxicity expressed as percentage (%) at different concentrations.
