# Supplementary material for: Prediction of Terpenoid Toxicity Based on a Quantitative Structure–Activity Relationship Model
Source: Foods. 2019 Dec 1;8(12):628. doi: 10.3390/foods8120628 (PMC6963511; doi:10.3390/foods8120628)
Supplement: Supplementary file 1 [file foods-08-00628-s001.pdf]

Table S1. Monoterpenic compounds toxicity expressed as percentage (%) at different concentrations.

Monoterpenoids	Concentration (μM)	Incubation times (min)*				
		20	40	60	80	100
p-Cymene	1	0.96	1.19	0.93	1.36	0.96
	10	15.54	18.56	13.97	17.85	17.91
	100	29.26	29.76	33.85	33.93	33.17
	200	72.16	67.56	68.56	73.15	75.07
(*R*)-(+)-Limonene	1	1.43	0.79	1.25	0.43	1.13
	10	24.79	24.88	31.99	27.50	28.46
	100	39.93	39.63	41.42	44.15	42.07
	200	54.37	55.17	59.52	59.47	55.99
(+)-Borneol	1	1.03	0.87	1.36	1.21	1.27
	10	6.38	7.71	10.06	11.23	9.97
	100	9.58	11.34	12.08	11.20	8.71
	200	12.82	15.15	15.98	19.82	16.81
Eucalyptol	1	1.81	1.85	1.83	1.38	1.84
	10	7.01	7.29	8.07	8.83	7.57
	100	7.37	12.93	11.11	11.85	11.97
	200	12.57	14.96	14.46	14.42	17.10
Geraniol	1	4.11	4.50	5.36	6.45	6.05
	10	19.03	18.65	17.32	18.44	18.87
	100	43.82	44.37	45.24	46.71	44.94
	200	68.17	69.19	70.53	68.08	69.82
Linalool	1	1.43	1.45	3.38	2.38	2.34
	10	14.06	15.63	18.12	19.68	21.74
	100	43.12	43.27	42.85	41.41	42.12
	200	77.52	79.43	79.63	76.46	76.91
Nerol	1	4.77	2.22	2.46	2.10	2.54
	10	14.57	16.31	17.66	12.35	13.92
	100	35.39	34.77	34.83	36.71	38.16
	200	53.40	51.78	48.50	52.09	51.95
α-Terpeniol	1	0.39	0.45	0.62	0.74	0.63
	10	16.04	16.15	16.91	16.08	16.54
	100	39.59	39.53	41.61	41.39	41.86
	200	60.52	60.54	61.62	58.81	60.03
β-Citronellol	1	2.76	3.19	2.31	2.32	3.13
	10	51.52	51.40	52.19	52.38	51.39
	100	63.03	61.28	62.09	62.18	62.07
	200	82.77	83.95	86.96	83.80	89.13
(-)-Menthol	1	3.08	2.66	2.71	1.90	2.31
	10	21.00	20.08	22.70	22.97	20.45
	100	25.75	25.80	24.04	24.23	25.71
	200	33.05	28.69	27.85	29.02	28.40
(*R*)-Carvone	1	3.78	2.12	2.24	3.49	2.37
	10	7.20	6.55	6.80	5.38	5.88
	100	16.63	15.77	21.27	23.35	21.85
	200	41.42	38.07	37.95	38.71	39.60

Citral	1	8.12	9.06	8.57	9.81	9.31
	10	9.29	10.86	10.42	10.14	9.83
	100	40.49	38.38	38.15	39.15	41.13
	200	69.97	69.45	68.67	67.27	64.41
(S)-Citronellal	1	7.24	9.49	9.68	7.30	7.40
	10	16.28	22.73	27.80	29.49	30.46
	100	57.68	60.13	59.54	60.00	59.56
	200	62.55	71.62	71.49	67.79	64.65
Geranic acid	1	18.13	20.17	20.97	20.07	20.93
	10	24.64	22.68	25.29	26.95	28.87
	100	48.35	44.49	47.16	48.44	49.34
	200	62.19	65.30	61.24	62.91	64.55
Linalool oxide	1	4.02	4.08	2.54	3.19	3.29
	10	4.69	8.66	10.84	8.19	7.87
	100	12.38	12.60	12.36	12.09	11.72
	200	16.87	18.12	22.15	21.91	21.03
(±)-α-Terpinyl acetate	1	9.91	11.44	11.27	12.15	10.04
	10	30.63	32.12	30.99	36.91	31.56
	100	53.71	55.66	53.03	53.50	53.49
	200	59.73	62.58	65.07	63.86	65.82

*The values are expressed as the means of three independent experiments.

Table S2. Sesquiterpenic compounds toxicity expressed as percentage (%) at different concentrations.

Sesquiterpenoids	Concentration (μM)	Incubation times (min)*				
		20	40	60	80	100
(-)-α-Cedrene	1	0.71	1.45	1.08	1.22	1.49
	10	13.63	16.20	26.22	25.99	22.02
	50	34.41	39.37	41.40	40.75	44.16
	100	50.56	54.77	57.29	62.03	61.64
(-)-α-Neoclovene	1	0.33	0.26	0.92	0.35	0.37
	10	9.11	10.33	11.62	12.94	8.89
	50	30.09	30.60	32.37	36.49	33.80
	100	49.21	45.91	47.19	46.04	40.31
β-Caryophyllene	1	0.62	0.83	1.48	1.11	1.46
	10	5.87	3.50	5.19	6.12	6.28
	50	12.33	12.55	15.81	16.49	16.19
	100	19.71	16.48	17.61	22.97	19.46
(+)-Valence	1	1.50	1.84	1.76	1.85	1.42
	10	5.42	4.75	4.68	4.25	5.04
	50	9.56	8.80	8.79	9.14	9.96
	100	13.42	14.82	13.38	13.41	15.93
Guaiazulene	1	0.93	0.94	1.09	0.73	1.55
	10	4.19	4.80	3.78	4.82	4.00
	50	7.91	8.52	9.15	11.89	13.13
	100	13.56	15.35	14.63	19.36	18.48
(*Z*)-Nerolidol	1	2.86	3.57	3.96	3.30	4.08
	10	22.61	24.44	29.69	31.20	31.42
	50	76.36	76.15	81.58	78.39	80.35
	100	96.00	94.47	96.63	97.39	95.41
(-)-α-Bisabolol	1	1.52	2.77	1.31	1.73	1.72
	10	22.97	22.83	28.89	27.23	25.44
	50	33.21	30.83	32.03	36.66	36.23
	100	47.24	49.83	51.06	54.03	53.90
(*E*,*E*)-Farnesol	1	3.77	3.21	4.98	3.72	3.41
	10	12.36	12.75	19.44	17.89	16.60
	50	52.33	49.86	53.24	57.41	53.77
	100	61.14	65.71	70.36	69.72	69.19

*The values are expressed as the means of three independent experiments.

Table S3. Norisoprenoids toxicity expressed as percentage (%) at different concentrations.

Norisoprenoids	Concentration (μM)	Incubation times (min)*				
		**20**	**40**	**60**	**80**	**100**
β-Ionone	1	4.45	4.09	4.98	4.81	5.64
	10	28.54	32.64	30.27	32.49	30.15
	50	39.46	37.91	42.41	43.92	42.90
	100	66.55	69.67	68.35	69.80	67.99
Geranyl acetone	1	5.10	5.81	7.01	7.03	7.09
	10	24.33	24.16	29.27	30.39	30.35
	50	58.74	61.83	60.86	61.36	62.37
	100	77.64	77.14	74.24	77.72	79.88
(±)-Theaspirane	1	0.36	0.74	0.72	0.41	0.48
	10	12.33	11.02	9.95	11.23	11.77
	50	20.99	19.08	22.64	23.93	21.92
	100	31.34	29.94	30.26	31.22	29.62

*The values are expressed as the means of three independent experiments.
